# Transforming diabetes prevention and care in South Africa: Proceedings and outcomes of the 2025 Diabetes Summit

**DOI:** 10.4102/phcfm.v18i2.5577

**Published:** 2026-08-05

**Authors:** Patrick Ngassa Piotie, Ndinda Makina-Zimalirana, Alexandra van Essche, Penelope Modipane, Herbert M. Makgopa, Mariam Hassen, Asanda Mbewu-Hlobo, Kibachio Joseph Mwangi, Melanie Pienaar

**Affiliations:** 1 School of Health Systems and Public Health, Faculty of Health Sciences, University of Pretoria, Pretoria, South Africa; 2 Department of Public Health, Medical School, Nelson Mandela University, Gqeberha, South Africa; 3 Department of Public Health and Family Medicine, Faculty of Health Sciences, University of Cape Town, Cape Town, South Africa; 4 Diabetes Alliance South Africa, Pretoria, South Africa; 5 School of Medicine, Faculty of Health Sciences, University of Pretoria, Pretoria, South Africa; 6 Department of Physiology, Faculty of Health Sciences, University of Pretoria, Pretoria, South Africa; 7 World Health Organization, Pretoria, South Africa; 8 School of Nursing, Faculty of Health Sciences, University of the Free State, Bloemfontein, South Africa

## Introduction: Reframing a national crisis

Diabetes has emerged as the leading cause of death in South Africa,^1^ surpassing human immunodeficiency virus (HIV), tuberculosis (TB) and other priority health conditions. Its rapid progression threatens the sustainability of the healthcare system and has broader social and economic consequences. The crisis has exposed systemic weaknesses, including fragmented responses, insufficient prevention, underinvestment, undiagnosed disease and inequitable access to care and technologies.

Diabetes also imposes a substantial economic burden on South African health. The estimated direct medical cost of diagnosed type 2 diabetes in the public sector was approximately R2.7 billion in 2018, increasing to R21.8 billion when undiagnosed cases are included, with projections suggesting costs could reach R35.1 billion by 2030 if current trends persist.^2^

The Diabetes Alliance, South Africa’s national civil society coalition for diabetes, brings together individuals living with diabetes, advocacy organisations, healthcare professionals, academic institutions, government stakeholders and industry partners to advance equitable access to diabetes prevention, treatment and care.

Since 2021, the Alliance has convened the biennial South African Diabetes Summit,^3^ a national platform for advocacy, multisectoral collaboration and policy dialogue that reflects a whole-of-society approach to addressing diabetes.

The 2025 Diabetes Summit, held from November 11–13, 2025, in Johannesburg, was co-hosted by the Diabetes Alliance in collaboration with the National Department of Health, the Gauteng Department of Health, the South African Medical Research Council, the University of Pretoria, the World Health Organization South Africa and the World Diabetes Foundation. The Summit, themed ‘Innovate for Impact: Transforming the Future of Diabetes in South Africa’, convened stakeholders from more than 40 organisations across government, academia, civil society and the private sector.

This report presents the key reflections from the Summit and their implications for South Africa’s diabetes response. Across all thematic areas – awareness and prevention, education, management and access to care, surveillance, research and innovation and governance – discussions underscored a clear message: Reversing the diabetes epidemic will require bold national reforms, stronger multisectoral coordination, a coherent national strategy and recognition of diabetes as a public health crisis.

## What the Summit revealed about South Africa’s diabetes burden: A nation at a tipping point

The Summit exposed systemic weaknesses that undermined South Africa’s ability to prevent, detect and manage diabetes effectively.

### Prevention without impact indicators

The Awareness and Prevention session revealed gaps in health literacy, stigma, language accessibility and community engagement. Despite significant activities, including community health worker (CHW)-led screening, radio messages in 11 languages and traditional folk media initiatives, South Africa lacks mechanisms to assess the impact of prevention. The participants emphasised the absence of monitoring systems for primordial prevention, health promotion policies and screening campaigns.

Prevention efforts are undermined by weak digital marketing regulations, inconsistent enforcement of food labelling policies, political–industry conflicts of interest and poor health and economic integration. These structural barriers create obesogenic environments that the health system cannot counter.

### An education ecosystem unprepared for the epidemic

The education session revealed significant skill gaps among health professionals, people with diabetes and communities. The absence of a structured national diabetes education programme, despite high rates of amputations, kidney failure and blindness, was identified as a major health system shortcoming.^4^ The scarcity of podiatrists and lack of recognition of Diabetes Nurse Educators severely limit the ability to prevent complications.^5,6^

### Fragmented and inequitable care

The Management and Access to Care session revealed delays in diagnosis, clinical inertia, limited access to essential technologies and public–private health sector inequities.^7,8^ Although new diabetes drugs and devices exist, public sector uptake remains slow. Individuals with diabetes often lack glucometers, strips and lancets. Psychosocial barriers, such as food insecurity and depression, worsen outcomes.

South Africa faces significant challenges in managing diabetes owing to the absence of a unified workforce strategy. The education system inadequately prepares clinicians for chronic care, and specialist shortages, including those of clinical psychologists, podiatrists and nurse educators, hamper comprehensive and multidisciplinary diabetes care. These workforce gaps exacerbate inequities, delay diagnosis and impede treatment intensification.

### Significant gaps in diabetes surveillance

Perhaps the most striking insight came from the surveillance session: South Africa is ‘data rich but information poor’. With paper-based records, non-interoperable datasets and insufficient indicators in the District Health Information System (DHIS), the country lacks the surveillance backbone required to understand and control the epidemic. The need for a national diabetes registry and an integrated digital infrastructure has been repeatedly emphasised.

### Innovation constrained by system failures

While technological advancements such as artificial intelligence (AI), biobanking and point-of-care (POC) diagnostics hold great promise, the Summit revealed that these innovations are significantly constrained by an unprepared system. For instance, AI models cannot be trained without high-quality data, POC devices cannot be scaled without connectivity and biobanking is constrained by insufficient funding.

Collectively, these findings portray a health system under immense stress, in which innovations, policies and programmes exist in isolation rather than in a coordinated, mutually reinforcing manner.

### Governance challenges and avoidable costs

The Summit highlighted that South Africa’s diabetes crisis extends beyond biomedical challenges and is rooted in systemic and governance failures. No central national mechanism coordinates the response to diabetes. Instead, responsibilities are dispersed across directorates, government departments and provincial structures, causing inefficiencies and missed opportunities. Summit participants stressed the need for decisive national leadership to enforce accountability and resource alignment.

The cost of fragmentation is high, with billions spent on amputation, dialysis, heart failure admissions and disability.^2,9,10^ However, prevention and early intervention remain underfunded. The Summit’s message was clear: Long-term economic and social losses far outweigh the investments required for a coordinated national response.

## Towards a coherent national response: Insights for designing a national diabetes strategy

South Africa’s National Strategic Plan (NSP) for the Prevention and Control of Non-Communicable Diseases (NCDs) (2022–2027) provides a framework for NCD prevention and care.^11^ The 90-60-50 cascade targets for diabetes and hypertension aim to ensure that 90% of adults know their glucose and/or blood pressure status, 60% of those diagnosed receive treatment and 50% achieve good control. These targets provide a clear pathway to improve screening, diagnosis, treatment and management.

Discussions at the 2025 Summit confirmed concerns from the 2023 Diabetes Summit Report that South Africa lacks the implementation framework to achieve the NSP’s 90-60-50 targets.^12^ Participants highlighted systemic deficiencies, underscoring the need for a National Diabetes Strategy to translate the NSP’s objectives into actionable, coordinated and measurable reforms.

Based on the Summit proceedings, a future National Diabetes Strategy should include the following:

### National alignment around clear strategic goals

The strategy should unify prevention, education, clinical management, surveillance and research under a single policy framework, aligned with the NSP yet operationalised through Specific, Measurable, Achievable, Relevant, Time-bound (SMART) indicators.

### A national diabetes education ecosystem

- Diabetes Self-Management Education and Support (DSMES) embedded in all levels of care.
- Recognition and deployment of Diabetes Educators.
- Expanded CHW capacity for screening, follow-up and culturally appropriate education.
- Foot-care training and podiatry integration.

### Strengthening health system architecture

- Multidisciplinary teams at the primary health centre (PHC) and district levels.
- Digital care pathways and e-prescribing.
- Integration with HIV, TB, Mother and Child Health and other high-burden programmes.
- Standardised clinical guidelines and person-centred care models.

### A national surveillance backbone

- A national diabetes registry.
- Standardised electronic medical records.
- Interoperable datasets to support AI and predictive analytics.
- Sentinel surveillance and periodic national surveys.

### Evidence-driven innovation

Innovative tools should be evaluated for cost-effectiveness and equity before national scale-up to ensure that digital and biomedical innovations reach underserved communities.

## A call for a national multisectoral coordination mechanism

Drawing on lessons from HIV governance, the Summit participants stressed that diabetes requires a mechanism to coordinate the government, civil society, academia, representatives of people with diabetes and the private sector. Such a platform would:

- Align efforts under the National Diabetes Strategy.
- Strengthen accountability and resource mobilisation.
- Facilitate multisectoral interventions addressing food environments, social and commercial determinants and health literacy.
- Conduct routine reviews of progress towards national targets.

Without such a mechanism, the national response remains fragmented and unable to scale up innovations or sustain reforms.

## Recognising diabetes as a national public health crisis

The Summit concluded: ‘Diabetes is the number one cause of death – not TB, not HIV – but diabetes’. Diabetes, as the leading cause of mortality, along with the pervasive systemic failures previously outlined, requires an urgent and decisive national response by declaring diabetes a public health crisis. This declaration emphasises the need to prioritise diabetes on the highest political agenda.

A national crisis declaration would unlock rapid-response financing, accelerate the implementation of the National Diabetes Strategy, enable the fast-tracking of technologies such as continuous glucose monitors (CGMs) for children with type 1 diabetes, strengthen surveillance and task-shifting reforms and signal a national commitment to saving lives.

Participants contended that the failure to declare a priority health crisis perpetuates suffering, avoidable complications and socioeconomic loss, thereby undermining South Africa’s broader response to NCDs.

## Conclusion: From momentum to mandate

The 2025 Diabetes Summit transcended conventional boundaries and served as a catalyst for South Africa to enhance its approach to an escalating national health crisis. The discussions highlighted significant systemic deficiencies while simultaneously presenting unparalleled opportunities for reform, innovation and multisectoral collaboration.

To achieve meaningful progress, South Africa must undertake the following actions:

- Declare diabetes a national public health crisis.
- Develop and implement a National Diabetes Prevention and Control Strategy.
- Establish a functional and mandated national multisectoral coordination mechanism with clearly defined structures and responsibilities that cascade to provincial, district and municipal levels.

Although the necessary tools, partnerships, and expertise exist, political will, decisive leadership and sustained commitment are essential to safeguard the health and dignity of millions of South Africans affected by diabetes mellitus.
